# Supplementary material for: MetaRibo-Seq measures translation in microbiomes
Source: Nat Commun. 2020 Jun 29;11:3268. doi: 10.1038/s41467-020-17081-z (PMC7324362; doi:10.1038/s41467-020-17081-z)
Supplement: Supplementary file 10 — Supplementary Data 7 [file 41467_2020_17081_MOESM10_ESM.zip › File2/Confidence_High_Taxonomy/430365_out.krona.html]

Javascript must be enabled to view this page.

members
magnitude
magnitudeUnassigned
count
unassigned
taxon
rank

430365\_out

6

superkingdom
6
2

6
phylum
1239

class
6
186801

186802
6
order

541000
family
6

1263
genus
6

species
1

SRS014979\_contig\_number\_34453
1638786


SRS015431\_contig\_number\_69435SRS019068\_contig\_number\_93845SRS146812\_contig\_number\_10618
40519
3
species


SRS097889\_contig\_number\_40678SRS143780\_contig\_number\_16092
2292375
2
species
